# Supplementary material for: End Sequence Analysis Toolkit (ESAT) expands the extractable information from single-cell RNA-seq data
Source: Genome Res. 2016 Oct;26(10):1397–410. doi: 10.1101/gr.207902.116 (PMC5052061; doi:10.1101/gr.207902.116)
Supplement: Supplemental Material [file supp_gr.207902.116_Supp_Materials.tar.gz › ESAT-esat_v0.1/doc/html/classbroad_1_1pda_1_1seq_1_1protection_1_1_multi_sample_scan_peak_caller-members.html]

ESAT: Member List


ESAT


- Main Page
- Related Pages
- Packages
- Classes
- Files

- Class List
- Class Index
- Class Hierarchy
- Class Members

All Classes Namespaces Files Functions Variables Enumerator Pages

- broad
- pda
- seq
- protection
- MultiSampleScanPeakCaller

broad.pda.seq.protection.MultiSampleScanPeakCaller Member List

This is the complete list of members for broad.pda.seq.protection.MultiSampleScanPeakCaller, including all inherited members.

|  |  |  |
| --- | --- | --- |
| allSamples | broad.pda.seq.protection.MultiSampleScanPeakCaller | protected |
| binomialWindowScores | broad.pda.seq.protection.MultiSampleScanPeakCaller | protected |
| closeFilterRejectWriters() | broad.pda.seq.protection.MultiSampleScanPeakCaller | protected |
| commandLineHasDebugFlag(String[] commandArgs) | broad.pda.seq.protection.MultiSampleScanPeakCaller | protectedstatic |
| commandLineOutDir(String[] commandArgs) | broad.pda.seq.protection.MultiSampleScanPeakCaller | protectedstatic |
| controlSamples | broad.pda.seq.protection.MultiSampleScanPeakCaller | protected |
| createFromCommandArgs(String[] commandArgs) | broad.pda.seq.protection.MultiSampleScanPeakCaller | protectedstatic |
| expressionData | broad.pda.seq.protection.MultiSampleScanPeakCaller | protected |
| FILTER\_REJECT\_DIR | broad.pda.seq.protection.MultiSampleScanPeakCaller | protectedstatic |
| genes | broad.pda.seq.protection.MultiSampleScanPeakCaller | protected |
| getCoordinateSpace() | broad.pda.seq.protection.MultiSampleScanPeakCaller |  |
| getOneRandomSamplePermutation() | broad.pda.seq.protection.MultiSampleScanPeakCaller | protected |
| getPeakBedFileName(SampleData sample, String outDir, String chrName) | broad.pda.seq.protection.MultiSampleScanPeakCaller | protected |
| getSingleSampleScanPeaks(SampleData sample, Gene gene) | broad.pda.seq.protection.MultiSampleScanPeakCaller |  |
| initializeFilterRejectWriters(String commonSuffix, String outDir) | broad.pda.seq.protection.MultiSampleScanPeakCaller | protected |
| isExpressed(Gene gene) | broad.pda.seq.protection.MultiSampleScanPeakCaller |  |
| isExpressedInAllControlSamples(Gene gene) | broad.pda.seq.protection.MultiSampleScanPeakCaller |  |
| logger | broad.pda.seq.protection.MultiSampleScanPeakCaller | protectedstatic |
| main(String[] args) | broad.pda.seq.protection.MultiSampleScanPeakCaller | static |
| mergePeaks(Collection< Annotation > peaks) | broad.pda.seq.protection.MultiSampleScanPeakCaller |  |
| MultiSampleScanPeakCaller(MultiSampleScanPeakCaller other) | broad.pda.seq.protection.MultiSampleScanPeakCaller | protected |
| MultiSampleScanPeakCaller(String sampleListFile, String bedFile, String chrSizeFile, int window, int step) | broad.pda.seq.protection.MultiSampleScanPeakCaller |  |
| MultiSampleScanPeakCaller(String expressionBamFile, String signalBamFile, String bedFile, String chrSizeFile) | broad.pda.seq.protection.MultiSampleScanPeakCaller |  |
| MultiSampleScanPeakCaller(String expressionBamFile, String signalBamFile, String bedFile, String chrSizeFile, int window, int step) | broad.pda.seq.protection.MultiSampleScanPeakCaller |  |
| numControls | broad.pda.seq.protection.MultiSampleScanPeakCaller | protected |
| numSamples | broad.pda.seq.protection.MultiSampleScanPeakCaller | protected |
| numSignals | broad.pda.seq.protection.MultiSampleScanPeakCaller | protected |
| random | broad.pda.seq.protection.MultiSampleScanPeakCaller | protected |
| setBinomialScore(boolean binomialScore) | broad.pda.seq.protection.MultiSampleScanPeakCaller |  |
| setCoordinateSpace(CoordinateSpace space) | broad.pda.seq.protection.MultiSampleScanPeakCaller |  |
| setExpressionScanPvalueCutoff(double expressionScanPvalCutoff) | broad.pda.seq.protection.MultiSampleScanPeakCaller |  |
| setExtraFields(boolean useExtraFields) | broad.pda.seq.protection.MultiSampleScanPeakCaller |  |
| setFilterByStrand(boolean useStrandFilter) | broad.pda.seq.protection.MultiSampleScanPeakCaller |  |
| setFirstReadTranscriptionStrand(boolean firstReadIsTranscriptionStrand) | broad.pda.seq.protection.MultiSampleScanPeakCaller |  |
| setLoggerLevel(Level level) | broad.pda.seq.protection.MultiSampleScanPeakCaller | protected |
| setPeakCutoffMostCommonReplicate(double maxPct) | broad.pda.seq.protection.MultiSampleScanPeakCaller |  |
| setPeakTrimQuantile(double trimPeakQuantile) | broad.pda.seq.protection.MultiSampleScanPeakCaller |  |
| setPeakWindowCountCutoff(double peakCountCutoff) | broad.pda.seq.protection.MultiSampleScanPeakCaller |  |
| setPeakWindowScanPvalCutoff(double peakScanPvalCutoff) | broad.pda.seq.protection.MultiSampleScanPeakCaller |  |
| signalSamples | broad.pda.seq.protection.MultiSampleScanPeakCaller | protected |
| sizeFile | broad.pda.seq.protection.MultiSampleScanPeakCaller | protected |
| stepSize | broad.pda.seq.protection.MultiSampleScanPeakCaller | protected |
| windowSize | broad.pda.seq.protection.MultiSampleScanPeakCaller | protected |
| writeSingleSampleScanPeaks(SampleData sample, String outFile, String chrName) | broad.pda.seq.protection.MultiSampleScanPeakCaller | protected |


---

Generated on Fri Aug 15 2014 15:11:03 for ESAT by  

 1.8.7
